# Use of stepwise lactate kinetics-oriented hemodynamic therapy could improve the clinical outcomes of patients with sepsis-associated hyperlactatemia

**DOI:** 10.1186/s13054-017-1617-1

**Published:** 2017-02-16

**Authors:** Xiang Zhou, Dawei Liu, Longxiang Su, Bo Yao, Yun Long, Xiaoting Wang, Wenzhao Chai, Na Cui, Hao Wang, Xi Rui

**Affiliations:** 1 Department of Critical Care Medicine, Peking Union Medical College Hospital, Peking Union Medical College and Chinese Academy of Medical Sciences, Beijing, 100730 China; 2 Department of Critical Care Medicine, The Affiliated Hospital of Qingdao University Medical College, Qingdao, Shandong Province 266000 China

**Keywords:** Lactate kinetics, ScvO_2_, Sepsis, Septic shock, Hemodynamic therapy

## Abstract

**Background:**

Setting lactate kinetics at >30% might improve the clinical outcomes of patients with sepsis-associated hyperlactatemia. The aim of this study was to explore the outcome benefits of stepwise lactate kinetics vs central venous oxygen saturation (ScvO_2_)-oriented hemodynamic therapy at 6 h as the protocol goal during early resuscitation.

**Methods:**

The relevant parameters and adverse events after different targets in 360 randomly assigned patients with sepsis-associated hyperlactatemia were recorded and compared.

**Results:**

Heart rate (HR) at 48 h in the ScvO_2_ group was higher than in the lactate kinetics group (105 ± 19 bpm vs 99 ± 20 bpm, *P* = 0.040). The liquid balance at 4 h, 12 h, and 24 h in the lactate kinetics group was larger than in the ScvO_2_ group (1535 (1271–1778) ml vs 826 (631–1219) ml, *P* < 0.001; 1688 (1173–1923) ml vs 1277 (962 − 1588) ml, *P* <0.001; and 1510 (904–2087) ml vs 1236 (740–1808) ml, *P* = 0.005), respectively. Mortality was higher in the ScvO_2_ group (27.9% vs 18.3%, *P* = 0.033), but there was no significant difference between the two groups in the length of stay in the ICU or mechanical ventilation. In terms of new onset organ dysfunction, there was a significant difference between the two groups in total bilirubin at 48 h and 72 h. Based on the 60-day survival curves, there was significantly more mortality in the ScvO_2_ group than in the lactate kinetics group (*X* ^2^ = 4.133, *P* = 0.042). In addition, fewer adverse events occurred in the lactate kinetics group.

**Conclusions:**

Stepwise lactate kinetics-oriented hemodynamic therapy can reduce mortality in patients with sepsis-associated hyperlactatemia compared with ScvO_2_-oriented therapy.

**Trial registration:**

National Institutes of Health Clinical Trials Registry, NCT02566460. Registered on 26 September 2015.

**Electronic supplementary material:**

The online version of this article (doi:10.1186/s13054-017-1617-1) contains supplementary material, which is available to authorized users.

## Background

Morbidity due to sepsis affects 56–91 people per 100,000/year, and the associated mortality remains at approximately 20% or even higher [1–3]. Early goal-directed therapy (EGDT) in the treatment of severe sepsis and septic shock strategies suggested by Rivers et al. in 2001 have had a far-reaching impact on the resuscitation of patients with severe sepsis or septic shock [4]. The EGDT strategies have been recognized and recommended by the Surviving Sepsis Campaign (SSC) since 2002 as the core concept of the SSC guidelines [5–7], with profound impacts on the behavior of physicians in critical care medicine worldwide.

However, recently, with the results of the ProCESS, ARISE and ProMISe trials having been published, clinicians have begun to doubt central venous oxygen saturation (ScvO2) as an important objective of EGDT during the resuscitation of patients with sepsis [8–10]. Lactate, as a product of the anaerobic metabolism of tissue, is considered closer to the real situation of poor tissue perfusion. In recent years, some studies have noted that lactate kinetics could predict the prognoses of patients with severe sepsis [11–15]. In an observational study Nguyen et al. reported that lactate kinetics of 10% might indicate a better prognosis in patients with sepsis, and that every 10% increase in lactate kinetics over 6 h could further reduce mortality [12].

The EMShockNet investigators performed a multicenter, randomized non-inferiority trial and demonstrated that there was no significant difference in mortality among patients with sepsis when they used lactate kinetics greater than 10% in 6 h, compared with ScvO_2_ ≥ 70% [14]. by Craig et al. found in a retrospective study that 36% lactate kinetics in 6 h could be used as a prognostic cutoff value [15]. We presumed that the reason why the superiority of lactate kinetics was not demonstrated might be that the target lactate kinetics was not set sufficiently high. Therefore, we set the target thresholds of lactate kinetics at 2 h up to 10%, 4 h up to 20%, and 6 h up to at least 30%, and we defined it as stepwise lactate kinetics-oriented hemodynamic therapy. We designed a single-center, randomized controlled study to assess stepwise lactate kinetics-oriented vs ScvO_2_-oriented hemodynamic therapy in early 6-h resuscitation among patients with sepsis-associated hyperlactatemia in terms of the benefits for outcome.

## Methods

### Study design and patient enrollment

The trial was performed in the Critical Care Department of Peking Union Medical College Hospital in China. Patients with sepsis-associated hyperlactatemia admitted to the hospital from January 2013 to December 2014 were enrolled. The research protocol was reviewed and approved by the Ethics Committee of Peking Union Medical College Hospital (PUMCH-S616) and was registered in the U.S. National Institutes of Health Clinical Trials Registry (NCT 02566460).

Patients were assessed according to inclusion criteria, which required that the patients be older than 18 years of age with confirmed or presumed infection, that they met two or more criteria for systemic inflammatory response syndrome [5–7, 16] and that there was evidence of refractory hypotension or serum lactate greater than 4 mmol per liter. Refractory hypotension was defined as systolic blood pressure below 90 mm Hg or mean arterial pressure (MAP) below 65 mm Hg after an intravenous fluid challenge of 20 ml or more per kilogram of body weight. Patients had to be enrolled in the study within 2 h of the earliest detection of the inclusion criteria and within 1 h of arrival in the ICU.

Patients were excluded if they met any of the following criteria: age younger than 18 years, pregnancy, an acute cerebral vascular event (Glasgow coma score <5), acute myocardial infarction or acute coronary syndrome, massive pulmonary embolism, status asthmaticus, primary diagnosed cardiac dysrhythmia, contraindication to central venous catheterization, active gastrointestinal hemorrhage, massive intra-abdominal infective focus without drainage, severe bronchopleural fistula, seizure, receiving chemotherapy or immunosuppressive therapy, or in the end stage of a disease. Non-inferiority testing was adopted in this study. Based on ScvO_2_, the mortality rate was approximately 25%, so α was set at 0.05, 1-β was set at 0.9, the non-inferiority value was 0.15, and the calculated sample size was 175 in each group. Randomization was performed with the use of a random number table. All of the patients or their legally authorized representatives provided written informed consent.

### Treatment

Eligible patients were randomly assigned in a 1:1 ratio to one of the two groups: the protocol-based lactate kinetics group or the protocol-based ScvO_2_ group. When these patients were admitted to the ICU, they were referred to a single critical care physician, who was assigned as the research investigator. Routine procedures for the treatment of shock in the ICU, such as intratracheal intubation and central venous catheterization with the tip of the catheter located in the superior vena cava, were performed. Central venous pressure (CVP) was measured continuously using a bedside monitor. Antibiotics were administered intravenously at the discretion of the attending clinician. After the baseline measurements, the patients were randomized into one of the two groups by opening an opaque, sealed envelope containing the randomization assignment.

Patients assigned to the ScvO_2_ group received chest or neck central venous catheterization capable of measuring continuous ScvO_2_ (Edwards Lifesciences, Irvine, CA, USA). Then, the patients were resuscitated following the EGDT protocol, which was used by Rivers et al. [4]. The protocol provides for sequential resuscitation to meet the thresholds of central venous pressure, followed by MAP and then ScvO_2_ within the first 6 h of ICU admission. First, either crystalloid or colloid could be administered to achieve a CVP of 8 − 12 mmHg. Second, if the MAP was below 65 mm Hg, norepinephrine was administered to maintain MAP ≥65 mmHg. If the MAP was above 90 mm Hg, vasodilators were administered until it was 90 mm Hg or lower. Finally, if ScvO_2_ was below 70%, red blood cells were transfused to achieve a hematocrit of at least 30%. If ScvO_2_ continued to be below 70%, dobutamine was initiated and titrated in attempts to achieve ScvO_2_ of at least 70%.

Patients assigned to the lactate kinetics group were treated according to the protocol of lactate kinetics-oriented therapy within the first 6 h of ICU admission. All of the patients in this group also accepted with central venous catheterization to monitor CVP, but they did not have ScvO2 monitored. They had similarly targeted thresholds in CVP, followed by MAP. However, blood lactate in the lactate kinetics group was monitored at a minimum of 1-h intervals:

$$ \mathrm{Lactate}\ \mathrm{kinetics} = \frac{\left({\mathrm{Lactate}}_{\mathrm{initial}} - {\mathrm{Lactate}}_{\mathrm{time}}\right)}{{\mathrm{Lactate}}_{\mathrm{initial}}}\times 100\%. $$

Lactate_initial_ was the blood lactate level when the patients were included in the study; lactate_time_ was the blood lactate level measured at relevant time points. We set the target thresholds of lactate kinetics at 2 h up to 10%, 4 h up to 20%, and 6 h up to at least 30%. After the lactate kinetics group achieved the resuscitation goals of CVP and MAP, if the lactate kinetics rate had not reached the intended target at the corresponding time point, red blood cells were transfused to achieve a hematocrit of at least 30%. If the lactate kinetics remained below the intended target at the corresponding time point after the hematocrit was at least 30%, dobutamine was initiated and titrated, in an attempt to achieve the target value. Lactate measurements were performed on venous whole blood samples using a blood gas analyzer (ABL90 FLEX, Radomater Medical, Copenhagen, Denmark).

After the 6 h of early intervention, the two groups continued to be observed at 12, 24, 48, and 72 h, and using the same treatment strategies. In addition to the hemodynamic treatment mentioned, a series of sepsis treatments based on the 2012 SCC guidelines were performed, including eliminating the foci, using antibiotics early, providing low-dose glucocorticoids, and controlling blood sugar.

To ensure patient safety, the study allowed the physician in charge to decide to adjust the treatment of patients according to the clinical situation. For example, in the ScvO_2_ group, the doctor in charge could decide at some point to check the patient’s lactate and calculate the lactate clearance rate. In the lactate kinetics group, physicians could also decide at some point to start ScvO_2_ monitoring. However, these patients had to meet the following conditions for at least one item: (1) systolic blood pressure (SBP) was difficult to maintain (SBP <90 mmHg) or decreased urine output (<0.5 ml/kg per h); (2) respiratory conditions continued to worsen (including respiratory rate, oxygen saturation or oxygen requirement), and arterial blood gas or mechanical ventilation conditions continued to deteriorate; or (3) consciousness continued to deteriorate. Once patients received the treatment of the other group, he or she was also considered to be included due to intention-to-treat selection.

### Outcome measurements

After the patients’ enrollment, case report forms (CRFs) were completed by the attending physicians according to the protocols. The primary endpoint was 60-day mortality. The secondary end points were ICU and hospital length of stay, ventilator-free days, and newly emerging organ failure. Other end points included the number of protocol goals achieved, administered treatments, and protocol-related adverse events. The protocol-related data were collected at baseline, at each hour for 6 h and then at 24, 48, and 72 h. All of the patients were followed up until 60 days. The protocol implementation and data collection stopped when the patients or their families wished to withdraw from the program or refused further treatment, or if the patient died.

### Statistical analysis

The data were analyzed using intention-to-treat principles. The count data were analyzed using the chi-square test or Fisher's exact method; for measurement data, if the two sets of data did not follow a normal distribution, they were described by medians (interquartile ranges), and we used the Mann-Whitney *U* test to compare the two groups. If they had a normal distribution, mean ± standard deviation was used, and the independent sample *t* test was adopted to compare the two groups. Sixty-day survival curves were plotted, and the mortality rates in both groups were analyzed using the Breslow survival test. All of the data analyses were performed using SAS software, version 9.2 (SAS Institute Inc., Cary, NC, USA).

## Results

### Baseline characteristics

**Fig. 1:**
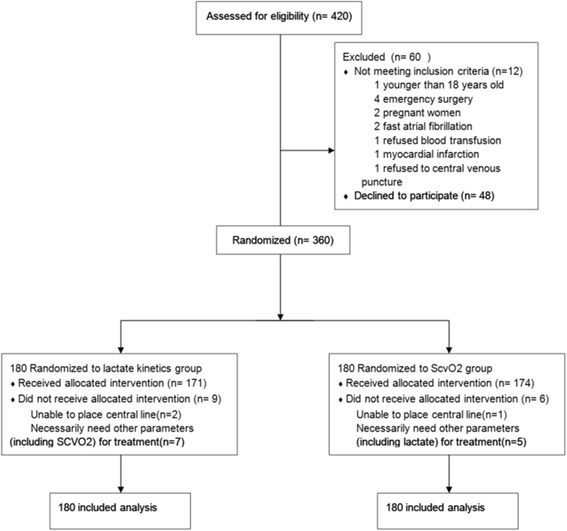
The flowchart of the enrollment of this study. *ScvO2* central venous oxygen saturation

Between January 2013 and December 2014, a total of 420 patients with sepsis met the inclusion criteria. Among these patients, 12 met the exclusion criterion, and 48 refused to participate in this study. Finally, 360 eligible patients were randomized into the lactate kinetics group (*n* = 180) and the ScvO_2_ group (*n* = 180). Among the 180 patients in the lactate kinetics group, an internal jugular or subclavian catheter could not be inserted in 2 patients, the original plan was not implemented in 7 patients during the trial, and 19 patients did not meet the planned target, despite the protocol having been strictly followed. Among the 180 patients in the ScvO_2_ group, a jugular or subclavian catheter could not be inserted in 1 patient, 5 patients did not have the original plan implemented, and 15 patients did not meet the original plan (Fig. 1).

There were no significant differences between the two groups in the baseline data. The sites of infection in the two groups all indicated that pulmonary infection (48.3% vs 47.7%, *P* = 0.916), severity of illness (APACHE II score 18 (14–30) vs 20 (14–31), *P* = 0.512), organ function (alanine aminotransferase (ALT), total bilirubin (TBIL), serum creatinine (SCr), and troponin (Tni)) and hemodynamic status (HR, MAP, CVP, lactate) did not differ significantly (Table 1).

**Table 1:** The baseline data for patients with septic shock who were enrolled in this study

	Lactate kinetics group	ScvO_2_ group	*P* value
	*n* = 180	*n* = 180	
Sex, male/female, *n*	95/85	106/74	0.289
Age, years	56 (44–66)	56 (40–67)	0.455
APACHE II score	18 (14–30)	20 (14–31)	0.512
WBC counts, ×10^9/L	12.9 (8.4–17.9)	12.0 (8.4–17.3)	0.957
Hemoglobin, g/L	101 (86–117)	105 (85–119)	0.217
Platelets, ×10^9/L	143 (85–191)	125 (74–196)	0.798
PT, s	15.4 (13.3–18.0)	15.1 (13.0–17.7)	0.547
APTT, s	36.2 (28.8–51.9)	35.6 (29.4–51.1)	0.519
ALT, U/L	106.0 (53.5–156.5)	78.0 (46.0–142.0)	0.161
TBIL, mmol/L	19.5 (11.6–37.2)	18.1 (10.7–41.9)	0.807
Albumin, g/L	27 (22–30)	27 (23–31)	0.109
sCR, mmol/dL	91.5 (65.3–163.5)	102 (65.5–182.5)	0.484
cTnI, mg/L)	0.42 (0.04–3.40)	0.29 (0.05–2.97)	0.635
HR, bpm	109 ± 25	114 ± 28	0.184
MAP, mmHg	83 (72–93)	87 (76–100)	0.113
Arterial lactate, mmol/L	5.8 (4.4–8.7)	5.6 (4.3–8.1)	0.156
Site of infection			
Lung, *n*	87	86	0.916
Intraperitoneal, *n*	58	60	0.822
Urinary system, *n*	1	1	1
Biliary tract infection, *n*	8	10	0.629
Skin and soft tissue, *n*	9	6	0.435
Blood infection, *n*	9	7	0.609
Other, *n*	8	10	0.629

*ScvO*_*2*_ central venous oxygen saturation, *APACHE* Acute Physiology and Chronic Health Evaluation, *WBC* white blood cells, *PT* prothrombin time, *APTT* activated partial thromboplastin time, *ALT* alanine transaminase, *TBIL* total bilirubin, *sCR* serum creatinine, *cTnI* cardiac troponin I, *HR* heart rate, *MAP* mean arterial pressure

### Treatment and vital signs

Of the 180 patients in the ScvO_2_ group, 5 required other indicators, such as lactate, to guide the clinical intervention. Therefore, the original plan was not implemented in these patients. The 6-h ScvO_2_ compliance rate was 98.9%. The average ScvO_2_ in the two groups was 77.7 ± 9.5%. Of the 180 patients in the lactate kinetics group, 7 required other indicators, such as ScvO_2_, to guide the clinical intervention. We recalculated the target achievement rate. The 2-h lactate kinetics target rate was met and the compliance rate above 10% was 83.9%; the 4-h lactate kinetics rate was 87.2%; and the 6-h target rate was 80.6%. The time course of ScvO2 in the ScvO2 group and lactate in the lactate kinetics group are shown as Additional file 1: Table S1.

There were no significant differences between the lactate kinetics and ScvO_2_ groups in the use of vasoactive drugs, inotropic drugs, or red blood cells. At 4 h, 12 h, and 24 h, more intravenous fluids were administered in the lactate kinetics group than in the ScvO_2_ group, with 1535 (1271–1778) ml vs 826 (631–1219) ml (*P* = 0.000), 1688 (1173–1923) ml vs 1277 (962–1588) ml (*P* = 0.000), and 1510 (904–2087) ml vs 1236 (740–1808) ml (*P* = 0.005), respectively (Table 2). There was no significant difference in the hemodynamic monitoring data for MAP, but HR in the ScvO_2_ group was higher at 48 h than in the lactate kinetics group (105 ± 19 bpm vs 99 ± 20 bpm, *P* = 0.040). CVP at 4 h, 6 h, and 12 h was greater in the lactate kinetics group than in the ScvO_2_ group (8 (6–10) mmHg vs 7 (5–9) mm Hg, *P* = 0.039; 9 (6–13) mmHg vs 8 (5–11) mmHg, *P* = 0.027; and 9 (7–11) mmHg vs 8 (6–10) mmHg, *P* = 0.041, respectively).

**Table 2:** Interventions in the ScvO_2_-oriented group and lactate kinetics-oriented group during the treatment process

	Lactate kinetics group	ScvO_2_ group	*P* value
	*n* = 180	*n* = 180	
RBC volume at 24 h, ml	144 ± 192	152 ± 193	0.705
Norepinephrine, %			
1st day	46.1% (83/180)	46.7% (84/180)	0.916
2nd day	38.9% (70/180)	40.6% (73/180)	0.747
3rd day	27.2% (49/180)	30.3% (54/180)	0.560
Norepinephrine, μg/kg/h			
	0 (0–4.46)	0 (0–7.75)	0.843
	0 (0–3.67)	0 (0–7.19)	0.1
	0 (0–0.57)	0 (0–2.45)	0.272
Dobutamine, %			
1st day	2.2% (4/180)	3.9% (7/180)	0.358
2nd day	4.4% (8/180)	3.3% (6/180)	0.586
3rd day	3.9% (7/180)	3.3% (6/180)	0.778
Dobutamine, μg/kg/h			
1st day	0 (0–0)	0 (0–0)	0.325
2nd day	0 (0–0)	0 (0–0)	0.263
3rd day	0 (0–0)	0 (0–0)	0.618
CVVH	26/154	36/144	0.163
Fluid volume, ml			
0–2 h	563 (259–922)	590 (288–960)	0.539
2–4 h	1535 (1271–1778)	826 (631–1219)	<0.001
4–6 h	1687 (1476–1929)	1255 (1008–1537)	0.358
6–12 h	1688 (1173–1923)	1277 (962–1588)	<0.001
12–24 h	1510 (904–2087)	1236 (740–1808)	0.005
24–48 h	465 (341–606)	424 (279–552)	0.071
48–72 h	278 (159–410)	284 (226–324)	0.502

*ScvO*_*2*_ central venous oxygen saturation, *CVVH* continuous veno-venous hemofiltration

### Primary and secondary outcomes

**Fig. 2:**
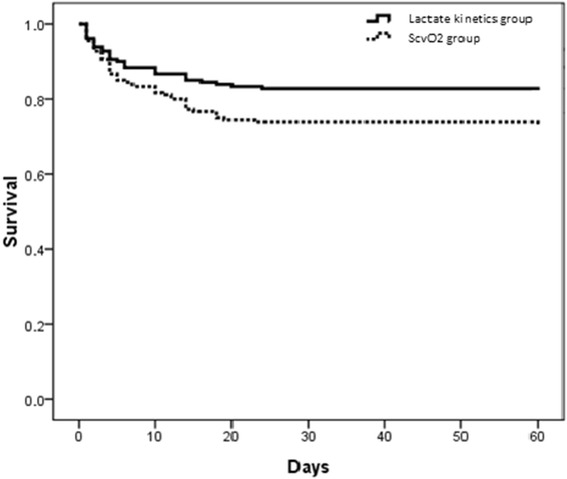
The 60-day survival curves in the central venous oxygen saturation (*ScvO*_*2*_)-oriented group and lactate kinetics-oriented group

Sixty-day mortality was higher in the ScvO_2_ group than in the lactate kinetics group (27.9% vs 18.3%, *P* = 0.033), but there was no significant difference between the two groups in the duration of mechanical ventilation while on the ICU. In terms of new onset of organ dysfunction, there was no significant difference in impairment of renal function in the lactate kinetics group compared with the ScvO_2_ group. There were significant differences in TBIL between the two groups at 48 h and 72 h (Table 3). Based on the 60-day survival curves, mortality was greater in the ScvO_2_ group than in the lactate kinetics group, with significant differences after Breslow testing (*X* ^2^ = 4.133, *P* = 0.042) (Fig. 2).

**Table 3:** Primary and secondary clinical outcomes in the ScvO_2_-oriented group and lactate kinetics-oriented group

	Lactate kinetics group	Central venous oxygen saturation (ScvO_2_) group	*P* value
	*n* = 180	*n* = 180	
Mortality at 60 days	33 (18.3%)	50 (27.9%)	0.033
Mechanical ventilation time, h	25 (14–97)	35 (16–106)	0.465
ICU stay time, h	68 (24–138)	66 (27–166)	0.348
Organ dysfunction			
Alanine aminotransferase, g/L			
0 h	106.0 (53.5–156.5)	78.0 (46.0–142.0)	0.161
24 h	125.0 (74.0–177.0)	118 (62.8–186.8)	0.844
48 h	114 (73.0–157)	109 (67–150)	0.47
72 h	113 (72–160.8)	109 (74–161.5)	0.725
Total bilirubin, mmol/L			
0 h	19.5 (11.6–37.2)	18.1 (10.7–41.9)	0.807
24 h	17.7 (11.7–32.4)	17.8 (10.7–38.7)	0.843
48 h	21.4 (13.3–35.5)	21.0 (12.6–50.8)	0.564
72 h	20.3 (13.1–33.7)	19.0 (11.8–40.2)	0.865
Serum creatinine, μmol/L			
0 h	91.5(65.3–163.5)	102(65.5–182.5)	0.484
24 h	79(59.0–128)	80(62.0–130)	0.443
48 h	81(58–117)	83(63.2–139.8)	0.217
72 h	72.5(53.8–111.5)	76(58–141)	0.123
Cardiac troponin I, μg/L			
0 h	0.42 (0.04–3.40)	0.29 (0.05–2.97)	0.635
24 h	0.14 (0.016–2.12)	0.12 (0.02–1.52)	0.766
48 h	0.30 (0.02–2.46)	0.21 (0.04–2.79)	0.745
72 h	0.19 (0.01–1.74)	0.15 (0.03–1.67)	0.61
Hemodynamic parameters			
Heart rate, beats per minute			
0 h	109 ± 25	114 ± 28	0.184
24 h	107 ± 24	112 ± 23	0.183
48 h	99 ± 20	105 ± 19	0.04
72 h	96 ± 24	99 ± 17	0.467
Mean arterial pressure, mmHg			
0 h	83(72–93)	87(76–100)	0.113
24 h	86 (74–96)	88 (75–99)	0.712
48 h	88 ± 15	85 ± 18	0.37
72 h	91 ± 12	87 ± 15	0.111
Central venous pressure, mmHg			
0 h	7 (5–8)	7 (6–8)	0.866
2 h	8 (6–10)	8 (7–10)	0.573
4 h	8 (6–10)	7 (5–9)	0.039
6 h	9 (6–13)	8 (5–11)	0.027
12 h	9 (7–11)	8 (6–10)	0.041
24 h	9 (7–11)	8.5 (7–11)	0.695
48 h	9 (7.3–10)	9 (7–10)	0.901
72 h	8 (6.3–10)	8 (6–9.7)	0.064

### Adverse events

Equal numbers of adverse events occurred in the two groups (Table 4). There were adverse events in four cases in the lactate kinetics group (2.2%), while in the ScvO_2_ group there were adverse events in 5 cases (2.8%). There was no statistically significant difference between the two groups (*P* = 1.000). Adverse events in the lactate kinetics group included one case of acute pulmonary edema, two cases of cardiac arrhythmia, and one case of catheter-related infections, while the adverse events in the ScvO_2_ group included one case of acute pulmonary edema, one case of acute myocardial infarction, and three cases of arrhythmia.

**Table 4:** The adverse event statistics in the ScvO_2_-oriented group and lactate kinetics-oriented group

	Lactate kinetics group	ScvO_2_ group	*P* value
	*n* = 180	*n* = 180	
Total number of adverse events, *n*/%	4/2.2%	5/2.8%	>0.05
Acute pulmonary edema, *n*	1	1	
Acute myocardial infarction, *n*	0	1	
Arrhythmia, *n*	2	3	
Cardiac arrest, *n*	0	0	
Pneumothorax caused by the puncture, *n*	0	0	
RBC allergy, *n*	0	0	
Catheter-related infections, *n*	1	0	

*ScvO*_*2*_ central venous oxygen saturation, *RBC* red blood cells

## Discussion

The main finding of this randomized, controlled trial was that stepwise lactate kinetics-oriented hemodynamic therapy could reduce mortality among patients with sepsis-associated hyperlactatemia, compared with ScvO_2_-oriented therapy. The therapeutic targets were reached in most patients during the first 6 h, which could indicate that the protocols were easily accepted and implemented. The lactate kinetics group had more fluid within the first 24 h, especially at 4 h, 12 h, and 24 h, but there were no differences between the two groups at 48 h or 72 h. The lactate kinetics group had a lower heart rate and less norepinephrine than the ScvO_2_ group, especially at 48 h. However, there was no difference in CVP between the two groups. More patients required dobutamine in the lactate kinetics group than in the ScvO_2_ group, but the difference was not significant. In addition, there was no difference in adverse events between these two groups.

An optimal resuscitation strategy for sepsis patients should have two characteristics: (1) it should be able to generate sufficient driving force for the specific clinical treatment so that it can better guide the clinical therapy, and (2) it should also be able to achieve effective resuscitation while avoiding overtreatment. The strategy recommended by EGDT [4] and the SSC bundles [2] has two central points – CVP and MAP, respectively – and the end point is ScvO_2_. This strategy was very clear in outlining the clinical path of resuscitation in patients with sepsis, and it has been widely accepted and promoted over the past 10 years.

The IMPreSS study group demonstrated that compliance with SSC bundles was independently associated with improvements in hospital mortality among patients with sepsis [17]. Since ProCESS, ARISE, and ProMISe were published, the EGDT strategy in sepsis resuscitation has once again aroused great controversy [8–10]. All three of these studies demonstrated that protocols using ScvO_2_ as a resuscitation goal do not offer a survival advantage in patients with early septic shock. However, many researchers have assumed that the low mortality in the usual-care groups in the three studies might have been due to these patients receiving pre-existing protocol-driven care, as outlined in the SSC bundles [18, 19]. In fact, any treatment in clinical practice must have a clear goal to be reached. The core of the dispute is not the validity of goal-directed therapy strategies but the question as to whether ScvO_2_ is the proper goal to guide early sepsis resuscitation.

The average enrollment ScvO_2_ levels, which were defined as the average levels when the patients were enrolled, were very different in the study of Rivers et al. compared to more recent studies, including our own. The average enrollment ScvO_2_ was 49% in the study of Rivers et al. However, the average enrollment ScvO_2_ levels in ProCESS, ARISE, and ProMISe were 71%, 72.7%, and 70%, respectively, while in our study, it was 72.1% [8–10]. This discrepancy is one of the most important differences between the study of Rivers et al. and our study, indicating that there was quite a large proportion of patients in the ScvO_2_ group who could not receive future resuscitation according to the protocols in these studies (approximately 69% of the patients in our study). In fact, Rivers et al. also reported that low ScvO_2_ usually indicates inadequate oxygen delivery or excessive oxygen consumption, but high ScvO_2_ did not exclude hypoperfusion in their study [4]. Pope and Textoris reported that not only did low ScvO_2_ suggest poor prognosis but that high ScvO_2_ was also associated with poor prognosis in patients with sepsis [20, 21]. Thus, it could be that ScvO_2_-oriented therapy could not promote resuscitation in more than 50% of patients with sepsis and could not prevent overtreatment.

It has been widely accepted that initial hyperlactacidemia is associated with greater mortality in patients with sepsis, and it has been regarded as independent of other organ failure indicators [11, 22–25]. Lactate kinetics and its kinetics, which reflect the dynamic changes in blood lactate, might more accurately reflect the quality of shock resuscitation and could predict the prognosis of patients better than many other methods that consist purely of fixed measurements of hemodynamic parameters [11–14]. Therefore, we believe that lactate kinetics and its kinetics is a good endpoint of goal-directed therapy for resuscitation in patients with sepsis. However, the optimal cutoff value for lactate kinetics to guide resuscitation in sepsis is controversial. In clinical situations, any treatment to improve tissue perfusion (including increasing oxygen delivery (DO_2_) or decreasing oxygen consumption (VO_2_)) can reduce blood lactate levels to a certain extent, that is, the inherent ability that reflects the effects of recovery during the resuscitation process. Therefore, an optimal lactate kinetics threshold must be sufficiently high to produce a driving force to propel the resuscitation strategy forward.

To date, lactate kinetics has been as the endpoint of goal-directed therapy in the resuscitation of patients with sepsis in three randomized, controlled clinical trials. The EMShockNet investigators reported non-inferiority in terms of reduction in hospital mortality among the group with lactate kinetics greater than 10% at 6 h and in the group with ScvO_2_ ≥ 70% at 6 h (17% vs 23%) [15]. Jansen et al. reported that hospital mortality in the lactate kinetics group reaching 20% every 2 h was not different compared with the conventional treatment group (33.9% vs 43.5%, *P* = 0.067) [24]. Tian et al. compared the results of 28-day mortality between lactate kinetics greater than,, 10% and 30%, respectively at 6 h and ScvO_2_ > 70%. The results were significantly lower in the 30% lactate kinetics group (28.6%) and the 10% group (36.4%) than in the ScvO_2_ group (63.2%) (*P* < 0.05) [25]. However, this study included only 62 cases, and 28-day mortality in the ScvO_2_ group was much higher than that in the present study (63.2%.), while Craig et al. reported in a retrospective study that resuscitation within 6 h and lactate kinetics of 36% could forecast the prognoses of patients with sepsis, and the cutoff value was set at 36% [14]. Therefore, in our goal-directed therapy protocol, after CVP and MAP reached the targets, based on the existing research results, the target lactate kinetics was set at 10%, 20%, and 30% at 2 h, 4 h, and 6 h, respectively. This stepwise strategy resulted in more active therapy for patients with sepsis, especially in early fluid resuscitation.

Stepwise target setting lactate kinetics in our goal-directed therapy protocol could not only promote resuscitation, but it could also prevent overtreatment. As we know, despite full resuscitation, improvement in tissue perfusion requires a certain length of time. Lactate kinetics of 30% might be reasonable for early 6-h resuscitation, but for a shorter time, such as 2 or 4 h, it might be too high and could lead to overtreatment. Therefore, the targets of lactate kinetics were set at 10%, 20%, and 30% at 2 h, 4 h, and 6 h, respectively, in our study to ensure that the targets could promote therapy during various stages of resuscitation and that overtreatment could be avoided at full steam. Serial blood lactate levels reflect both lactate production and clearance [26, 27]. We could see that the lactate kinetics group achieved the more active therapy strategy, including more fluid infusion at 4 h, 12 h and 24 h, but there were no significant differences in the CVP levels, the ratio of applications, or the dosage of vasoactive and inotropic drugs between the two groups. Furthermore, there was no significant difference in fluid balance at 48 and 72 h between the two groups.

There were several limitations to this study. First, it was a single-center study. A larger, more rigorous multicenter study could further confirm our conclusions in the future. Second, this study was a non-blinded, randomized, controlled study. Therefore, this study used a rigorous method of randomization, rigorous training and inspection procedures to ensure that compliance with each step was maintained at a relatively high level (greater than 90%), thus minimizing the potential for errors. Third, this study explored only patients with definite diagnoses of sepsis, but patients with potential shock who were not diagnosed have not been studied. Early identification and resuscitation in these patients could also be a very critical topic in sepsis therapy. Fourth, there were significant differences between recent trials on EGDT and the Rivers trial. Patients were already resuscitated by some method (initial ScvO_2_ values or lactate values were comparable to those values after hours of therapy in the Rivers trial). The moment of starting resuscitation will have different impacts on outcome, and also importantly, the interpretation of several goals will be different.

## Conclusions

In summary, stepwise lactate kinetics target-oriented therapy could reduce mortality among patients with sepsis, compared with ScvO_2_-oriented protocolized therapy (≥70%). Lactate kinetics could become one of the indicators of resuscitation. However, it still requires large-scale and multicenter clinical studies to verify our findings.

## Additional file

Additional file 1: Table S1. Time course of ScvO2 in the ScvO2 group and of lactate in the lactate kinetics group. (DOCX 13 kb)

## Abbreviations

ALT: Alanine aminotransferase
CRF: Case report forms
cTnI: Cardiac troponin I
CVP: Central venous pressure
DO2: Oxygen delivery
EGDT: Early goal-directed therapy
HR: Heart rate
MAP: Mean arterial pressure
RBC: Red blood cells
SBP: systolic blood pressure
SCr: Serum creatinine
ScvO2: Central venous oxygen saturation
SSC: Surviving Sepsis Campaign
TBIL: Total bilirubin
Tni: Troponin
VO2: Oxygen consumption WBC, White blood cells
