# Supplementary material for: Use of stepwise lactate kinetics-oriented hemodynamic therapy could improve the clinical outcomes of patients with sepsis-associated hyperlactatemia
Source: Crit Care. 2017 Feb 16;21:33. doi: 10.1186/s13054-017-1617-1 (PMC5312433; doi:10.1186/s13054-017-1617-1)
Supplement: Additional file 1: Table S1. — Time course of ScvO2 in the ScvO2 group and of lactate in the lactate kinetics group. (DOCX 13 kb) [file 13054_2017_1617_MOESM1_ESM.docx]

**Additional file 1: Table S1**. The time course of ScvO2 of ScvO2 group and lactate of lactate kinetics group

|  | **Lactate value of lactate kinetics group** | **ScvO2 value of ScvO_2_ group** |
| --- | --- | --- |
|  | n=180 | n=180 |
| 0h | 5.8（4.4-8.7） | 79.0（71.0-84.0） |
| 2h | 4.4（3.1-6.5） | 77.0（70.0-82.0） |
| 4h | 3.1（2.1-4.7） | 78.0（74.0-82.0） |
| 6h | 2.1（1.4-3.4） | 78.0（76.0-84.0） |
| 12h | 1.5（1.0-2.6） | 78.0（74.0-82.0） |
| 24h | 1.2（0.8-1.9） | 79.0（71.0-84.0） |
| 48h | 0.9（0.7-1.6） | 74.0（68.0-80.0） |
| 72h | 1.0（0.8-1.6） | 74.0（68.0-81.0） |
